# Hepatitis C virus E1 protein-specific linear B-cell epitopes, pan-genotype reactivity, and functional relevance

**DOI:** 10.3389/fimmu.2026.1745299

**Published:** 2026-03-20

**Authors:** Babu E Preedia, Yuki Haga, Ranjit Ray

**Affiliations:** 1 Department of Internal Medicine, Saint Louis University, Saint Louis, MO, United States; 2 Department of Molecular Microbiology and Immunology, Saint Louis University, Saint Louis, MO, United States

**Keywords:** antibody response, B-cell epitope, E1 protein, ELISA, envelope glycoproteins, genotypes, hepatitis C virus, neutralizing antibody

## Abstract

**Background:**

Hepatitis C virus (HCV) infection poses a major global risk for chronic liver disease, underscoring the critical need for prevention. Our previous studies demonstrated that HCV-modified soluble E1/E2_F442NYT_ envelope glycoproteins delivered from an mRNA-lipid nanoparticle (LNP) vaccine platform induce immune responses *in vitro* in human cells and *in vivo* in immunized mice. These findings prompted us to analyze the sE1-derived induction of broadly cross-genotype HCV protective antibody response.

**Methods:**

Analyses for the evaluation of linear B-cell epitopes, specifically antibody elicitation by genotype 1a E1 mRNA-LNP immunization, were performed using a panel of E1 overlapping peptides. HCV pseudotype neutralization, interruption by antibody binding to epitope regions, and association between E1/E2 were biochemically analyzed for the impairment of known E2 receptor-binding functions, including surrogate virus neutralization.

**Results:**

Binding inhibition-based ELISA identified peptides having a distinct reactivity pattern of immune mouse sera, revealing four linear regions (Groups A–D). One of the peptides (Group A, P1) corresponded to an antigenic region previously characterized as a key linear B-cell epitope. HCV pseudotype neutralization assay using rabbit anti-P1 peptide serum demonstrated multi-HCV genotype neutralization. The relatively conserved linear B-cell epitope was found to localize in the α1 helix region, closer to the N-terminal ectodomain of E1/E2 major receptor-binding area. However, a much-conserved P2 epitope (Group D), with residues reported to interplay with the tight junction protein claudin-1 and scavenger receptor B type I, induced a detectable HCV pseudotype neutralization. Interruption by antibody binding to the P1 and P2 regions may impair intimate association between E1/E2, leading to the impairment of known E2 receptor-binding functions, including virus neutralization activity. Surface plasmon resonance suggested similar antibody binding affinity of the two rabbit antibodies (~10^−8^ M) to the unmodified E1/E2. The Group B peptide awaits further characterization for HCV functional inhibitory roles, while the Group C peptide is characterized as a putative fusion peptide and involved in cross-talk with E2.

**Conclusions:**

Collectively, the findings identified novel B-cell epitopes of E1 glycoprotein cross-reactive among HCV genotypes and may serve as a promising candidate to include for a rational design of a pan-genotypic HCV vaccine.

## 1 Introduction

Hepatitis C virus (HCV) infects over 50 million individuals globally and poses a major risk for chronic liver disease and hepatocellular carcinoma (HCC). Direct-acting antivirals (DAAs) constitute the frontline of HCV therapy. DAAs are effective in curing HCV infection, although the high cost and challenges in delivering to people in developing countries make it difficult to use worldwide (1), underscoring the critical need for a prophylactic HCV vaccine development. HCV envelope glycoproteins E1 and E2 are the key targets for vaccine development due to their role in viral entry and potential in eliciting protective immunity (2, 3). Our previous study demonstrated that modified sE2 envelope glycoprotein delivered via an mRNA-lipid nanoparticle (LNP) platform can induce immune responses both *in vitro* in human peripheral blood mononuclear cells (PBMCs) and *in vivo* in immunized mice (4). Notably, sE1 immunization elicited higher antibody titers and T cell-associated cytokine responses compared to sE2 or sE1/sE2 immunization (5). Additionally, the incorporation of sE1 with sE2_F442NYT_ (sE1/sE2_F442NYT_) demonstrated enhanced immunogenicity, suggesting the potential to advance HCV vaccine development. An immunoregulatory role appeared to be induced by the unmodified sE2 and extended to the sE1 glycoprotein-specific response in a candidate vaccine. The findings also prompted us to focus on the sE1-derived epitopes capable of inducing broadly cross-reactive and neutralizing antibody responses (6).

Serum cytokine analysis by ELISA demonstrated that sE1 vaccination induced a T-helper 1 skewed immune response, characterized by enhanced interleukin-2 and interferon-γ. The sE1-immunized mice showed a significant reduction in virus titers, indicating a protective immune response to the surrogate challenge infection (5). A strong neutralization of HCV chimeras was also noted using recombinant E1-specific mouse antiserum (7).

HCV E2 has been extensively characterized, whereas E1 remains relatively less studied. E1 promotes fragile conformation in association with E2 (8). Structural and molecular biology-related studies projected E1 contact points with E2, including four E1 amino acid residues (Y201, T204, N205, and D206 (9)), and 11 different amino acid residues of E2. The extended helical region in E1 (256–288) is essential for both fusion and E1/E2 heterodimerization (10). Additionally, a critical monomer disulfide bond connects E1 and E2 for correct E1/E2 folding and function. Here, we took advantage of the sE1 ectodomain to interrogate the identified potential B-cell functions for HCV inhibitory activity. Synthetic peptides derived from the HCV E1 region were shown to be highly conserved among the published virus genotypes. Thirteen out of 18 HCV-positive sera (72%) showed seroreactivity via ELISA (11). Therapeutic vaccination of chronically infected chimpanzees with the HCV E1 protein induced T-helper cell immune responses and antibodies (12), which were rarely seen in patients or chimpanzees with chronic hepatitis C (13–15).

We first reported that the P1 and P2 of the HCV E1 protein represent two potential linear B-cell epitopes (16). Subsequent examination suggested that the antisera raised against these two peptides display a strong ELISA titer, although they were not highly efficient in surrogate virus neutralization (17). The P2 represented the most conserved 10 amino acid stretch within the C-terminal region of the E1 protein derived from several HCV isolates. Productive folding of the major HCV spike E2 protein is assisted by E1 (18). The homology within the N-terminal region of E1 suggested that the domain plays a major role in E1/E2 interaction and folding of the envelope glycoproteins (19, 20). The recognition of sE1/sE2 proteins by heterodimer-specific broadly neutralizing antibodies, such as AR4A and AR5A, indicated the recognition of a native E1/E2 antigenic integrity (21). The AR4A binding depended on the presence of both subunits. Neutralizing antibodies were identified that target individual E1 and E2 subunits. Antibodies with an extraordinary breadth of neutralization were identified that depend on intact E1/E2 heterodimers for recognition (22–24). Vaccine antigens that retain native E1/E2 antigenic features may thus hold quaternary epitopes that can facilitate the induction of broadly neutralizing antibodies, although the design of such antigen preparation has proven challenging (25–27). Residues of the E1/E2 interface were highly conserved across HCV genotypes, as was the epitope of bound heterodimer-specific antibody AR4A that fell exclusively on E2. Thus, the epitopes within E1 that contribute to supporting E2-mediated immune responses remain to be characterized. The E1 epitope characterization is essential for understanding and enhancing E1/E2 targeted immunity, maximizing the protective capacity and breadth of an HCV vaccine.

In the present study, we characterized the potential B-cell epitopes of HCV sE1, validated them using computational B-cell prediction tools, assessed functional activity from the neutralization of peptide-specific immune sera, and evaluated their potential role in inhibiting viral entry through CD81 or scavenger receptor class B type I (SR-B1) receptor. Additionally, we assessed the relationship between epitope conservation and neutralization activity. Finally, limited structural analysis from the available information helped us to understand the potential for conformational changes induced by the binding of E1 and linear epitope-specific antibodies as a bystander effect on E2 functional relevance.

## 2 Materials and methods

### 2.1 Cell lines

Human hepatocellular carcinoma cell line Huh7.5 and human embryonic kidney 293T (HEK293T) cells were used in the study. Both the cells were maintained in Dulbecco’s modified Eagle medium (DMEM; Gibco, Green Island, New York, #11995040) supplemented with 10% fetal bovine serum (FBS) and 1% penicillin/streptomycin (Gibco, #15140122). Additionally, the HEK293T cell culture medium was supplemented with 1% nonessential amino acids.

### 2.2 Antisera to peptides and proteins

Rabbit antisera to peptide 1 (SLU22) and peptide 2 (SLU23) were generated using the peptides conjugated to keyhole limpet hemocyanin at the N-terminal regions (Cocalico Biologicals, Reamstown, PA, USA) as described previously (17). Fab fragments from SLU22 and SLU23 were generated using a Micro Preparation Kit (Pierce, Dalas, Texas) following the supplier’s protocol for surface plasmon resonance (SPR).

Sera from BALB/c mice immunized intramuscularly with sE1, sE2, sE2_F442NYT_ mRNA-LNP, or empty-mRNA-LNP as controls were used (28). Equal volumes of five mouse sera were pooled in each group and used as mentioned in the analyses.

### 2.3 mRNA-LNP candidate vaccine encoding HCV envelope glycoproteins

The mRNA candidate vaccines were generated as previously described (4). Briefly, the mRNA constructs were synthesized encoding codon-optimized HCV (genotype 1a/H77C) envelope glycoproteins using sE1 (aa 193–351), sE2 (aa 386–660), and sE2_F442NYT_ mutated at the indicated site. HCV sE2 and sE2_F442NYT_ proteins were examined and compared for the inserted glycosylation site at residue F442 after PNGaseF treatment at the Washington University Proteomics Shared Resource, St. Louis. The proteins were analyzed following constitutive deamidation of the residue and variable deamidation of other asparagine residues in the sequence. The mRNAs from HCV sE1, sE2, or sE2_F442NYT_ were generated and purified. The ethanolic lipid mixture comprising an ionizable cationic lipid, phosphatidylcholine, cholesterol, and polyethylene glycol-lipid was rapidly mixed with an aqueous solution at pH 4.0 containing cellulose-purified N1-mΨ *in vitro*-transcribed mRNAs for encapsulation into LNP. RNA-loaded particles were characterized by size, surface charge, encapsulation efficiency, and endotoxin content and stored at −80 °C at an RNA concentration of 1 μg/μL of loaded particles or an equivalent total lipid concentration for empty particles (29). The mean hydrodynamic diameter of mRNA-LNP was ~80 nm, with a polydispersity index of 0.02 to 0.06 and an encapsulation efficiency of ~95%. Two or three batches from each mRNA-LNP formulation were used in the study.

### 2.4 E1 peptide recognition by sE1-mRNA-LNP immunized mouse sera

Corning 96-well EIA/RIA assay microplate was coated with BEI peptides (2 µg/well) in carbonate buffer overnight at 4°C. The plates were washed five times with 0.05% Tween-20 in phosphate-buffered saline (PBS) and blocked with protein-free blocking buffer (Pierce) for 2 h at room temperature. Previously titrated sE1 immunized mouse serum was diluted at 1:400, added to the peptide-coated wells, and incubated overnight at 4°C. The plates were washed and incubated with goat anti-mouse antibody (1:3,000) for 1 h at room temperature. The color reaction was developed by the 3,3',5,5' tetramethylbenzidine (TMB) substrate and stopped by the addition of sulfuric acid, and absorbance was measured at 450 nm. The competition assay was performed similarly, except the plates were coated with HCV E1 (Sino Biological, Paoli, Pennsylvania) protein. The sE1 immunized serum was preincubated with the corresponding peptides before incubation to assess the inhibition of binding.

### 2.5 Identification of B-cell epitopes within selected peptides

The identification of linear B-cell epitopes was carried out using the Immune Epitope Database (IEDB) B-cell epitope prediction tool, BepiPred 2.0, which is based on a Random Forest algorithm. The peptide sequence of interest was submitted as a query, and upon completion, the predicted linear B-cell epitope residues were highlighted along the sequence. Since the IEDB BepiPred 2.0 is integrated within the comprehensive Immune Epitope Database platform, it allows combining epitope prediction with a database of experimentally validated epitopes. For further confirmation, DTU Health Tech BepiPred 2.0 was used, which provides a focused prediction based on sequence and structural features (30).

### 2.6 Analysis of peptide conservancy and biophysical properties

The conservancy of the identified peptides was analyzed across multiple HCV genotypes, including 1a (H77 and H72), 1b34, 1b58, 3.1.2, 4.2.2, and 5.2.1, using the Jalview software (30) and the IEDB conservancy tool (31). The aligned sequences were loaded as FASTA files, and multiple sequence alignment (MSA) was performed. Conservation scores were calculated and annotated below the alignment with graphical bars. Additionally, the Zappo color scheme was applied to visualize the physicochemical properties of the residues. The IEDB immunoinformatics tool was used for further sequence conservation visualization.

### 2.7 HCV pseudoparticle neutralization assay

HCV pseudoparticles (HCVpp) were generated by co-transfection of 2 μg pNL4.3.Luc.R-E plasmid, 0.5 μg pAdvantage plasmid in 250 μL into HEK293T cells Opti-MEM medium, and 1 μg plasmids expressing E1E2 from HCV genotypes 1a (H77C), 1a (H72), 1b (1b58), 1b (34), 3 (3.1.2), and 4 (4.2.2) available in our laboratory or obtained from Justin Bailey (Johns Hopkins University School of Medicine, Baltimore, MD, USA) using Lipofectamine 2000 (Invitrogen, Madison, Wisconsin) (31). The medium was changed after overnight incubation. The supernatants containing HCVpp were harvested 72 h post-transfection and filtered through a 0.45-μm pore size nitrocellulose membrane. For the HCVpp neutralization assay, Huh7.5 cells (1.8 × 10^4^) per well were placed in alternate wells of a 96-well white opaque tissue culture plate and incubated overnight at 37°C. The following day, HCVpp were mixed with or without immunized rabbit sera (SLU22 and/or SLU23) at indicated dilutions and incubated for 1 h at 37°C before adding to Huh7.5 cells for 6-h incubation at 37°C. Neutralization analysis was focused on limited low dilutions (e.g., 1:10 or 1:20) since only lower dilutions showed a defined neutralization titer. The medium was replaced and washed with PBS. Cells were lysed after 72 h with lysis buffer (EI53A, Promega), and 100 μL of luciferase substrate (ONE-Glo™ EX, Promega, Madison, Wisconsin) was added to each well. Luciferase activity was measured in relative luminescence units (RLU) using a GloMax luminometer (Promega). The percentage of neutralization was calculated as [1 − (RLU-sample/RLU-untreated)] × 100, with the untreated control RLU values representing the average of triplicates. All the assays were performed in the presence of known positive and negative samples. The results are presented using prior titrated samples showing a linear decrease at serial dilutions.

### 2.8 Surface plasmon resonance

The binding affinity and kinetic measurements of SLU22 or SLU23 Fab interaction with E1/E2 antigen were performed on Biacore S200 (Cytiva, Freiburg, Germany) using a CMD200M SPR sensor chip (XanTec, Dreieich, Germany) at 25°C. The immobilization of Fab onto a CMD200M chip was performed using amine coupling kit (Cytiva, USA) in which the chip was activated by 100 mM *N*-hydroxysuccinimide (NHS) and 400 mM 1-ethyl-3-(3-dimethylaminopropyl)carbodiimide hydrochloride (EDC) mixed 1:1 in 10 mM Hepes, pH 7.4, 150 mM NaCl (buffer A) for 400 s, followed by the immobilization of Fabs in 10 mM sodium acetate, pH 4.0, at 50 µg/mL for 3,000 RU in flow cells 2 and 3, respectively; flow cell 1 was used as a blank reference. Unreactive esters were quenched with a 400-s injection of 1 M ethanolamine-HCl, pH 8.5. Buffer A was used to wash off nonspecifically bound Fab; next, with PBS, pH 7.4, 0.05% Tween-20, 0.2% bovine serum albumin (BSA) (buffer B) was used overnight from the sensor chip surface to obtain a homogenous surface and block the non-specific interactions. A twofold serial dilution of the E1/E2 antigen (200 nM) was prepared in buffer B. The antigen at different dilutions was then injected onto the chip surface using a multi-cycle program in which the lowest to highest concentrations, including three injections of buffer before the lowest non-zero concentration for signal stabilization, were used with every step of regeneration by 10 mM glycine buffer, pH 2.7, 500 mM NaCl. For each concentration, data collection was performed at 120 s for association time and 300 s of dissociation time at a flow rate of 20 μL/min. The affinity and kinetic analyses were processed in the Biacore S200 Evaluation v1.1 software (Cytiva, USA), including reference subtraction. The data were fitted to a 1:1 Langmuir model to derive *k_a_* (“*k_on_*”, association rate constant), *k_d_* (“*k_off_*”, dissociation rate constant), and KD (equilibrium dissociation constant) values. The experiments were repeated twice.

### 2.9 FACS analysis

Huh7.5 cells were harvested using a non-enzymatic dissociation method with PBS containing 5 mM EDTA. The purified E1/E2 protein (generated from CHO cells) was preincubated with or without SLU22 and/or SLU23 at a 1:10 dilution at 37°C for 2 h. Cells (2.5 × 10^5^) were incubated with the E1/E2 antibody mixture at 4°C for 1 h in PBS containing 1% FBS. Cells were washed twice and stained with E2-specific AP33 monoclonal antibody (Sigma, Saint Louis, Missouri) conjugated with fluorescein isothiocyanate (FITC) (Biotin FITC labelling kit) for 45 min on ice. Subsequently, cells were washed and fixed in 1% formaldehyde. E1/E2 cell binding and inhibition were quantified via flow cytometry.

### 2.10 sE1/sE2 binding to hCD81 and SR-B1

Corning 96-well EIA assay microplate was coated with human CD81 protein (R&D, Minneapolis, Minnesota) at 1 µg/well or SR-B1 (Sino Biological) at 500 ng/well in carbonate buffer. After overnight incubation at 4°C, the plate was washed five times with 0.05% Tween-20 in PBS and blocked with protein-free blocking buffer (Pierce) for 2 h at room temperature. Meanwhile, tubes were prepared containing the following protein/antibody mixtures: E1/E2 protein (500 ng), E1/E2+SLU22, E1/E2+SLU23, and combinations of E1/E2+SLU22+SLU23 at 1:10 and 1:20 dilutions, respectively. All mixtures were placed at 37°C for 2 h and transferred to specific antigen-coated wells for further incubation overnight at 4°C. The wells were washed five times following incubation and incubated with E2-specific monoclonal antibody AP33 (Sigma) at a 1:1,500 dilution in PBS at 4°C overnight. After washing, an Horseradish peroxidase (HRP)-conjugated secondary anti-mouse IgG antibody was added and incubated for 1 h. The TMB substrate solution was added to each well, the reaction was stopped after 30 min by the addition of sulfuric acid, and the color was measured at 450 nM.

### 2.11 Statistical analysis

Statistical analyses were performed using the GraphPad Prism software. Data are presented as mean ± SD, with the number of biological replicates (n). Technical replicates are averaged before analysis. Comparison among the multiple groups was conducted using a two-way or one-way ANOVA followed by Tukey’s or Dunnett’s *post hoc* test. A *p*-value < 0.05 was considered statistically significant.

## 3 Results

### 3.1 Mouse sera immunized with HCV Env-mRNA-LNP recognize E1-specific linear B-cell epitopes

Pooled immune mouse sera elicited by E1-mRNA-LNP immunization from HCV genotype 1a were evaluated for epitope specificity. Sera were tested against a panel of 23 E1-specific peptides corresponding to HCV genotype 3a (BEI Resources, NIAID, Maryland). To minimize redundancy of overlapping peptides, only the odd-number peptides were selected for screening. The immunized mouse sera exhibited a strong reactivity toward BEI peptides 1, 3, 5, 7, 9, 11, and 13, indicating the presence of B-cell epitopes predominantly located within the N-terminal region of the E1 protein. Notably, BEI peptides 1–3 shared an overlapping 18 amino acid region spanning E1 amino acid (aa) residues 206–223, corresponding to the N-terminal domain (NTD) of the protein. A relatively less binding site was detected for BEI peptides 15, 19, 21, and 23. Native E1 protein anchors on the HCV surface through the C-terminal aa residues 353–383. Our immunizing antigen excluded the C-terminal transmembrane domain of the sE1. Similarly, BEI peptides 7, 8, and 9 overlap aa residues 244–254, a region located between NTD and the putative fusion peptide. Reactivity of the E1 peptide-immunized mouse sera pool appeared in the following order: BEI peptides 9 > 7 encompassing the overlapping sequences in peptide 9 (amino acid residues 243–259, TPTVAVRYVGATTASIR), inducing the antibody. Some of the amino acid residues in the N-terminal region were variable with the vaccinating mRNA-LNP genotype 1a (amino acid residues 232–248: N-terminal region TVATRDGRLP sequence).

**Figure 1:**
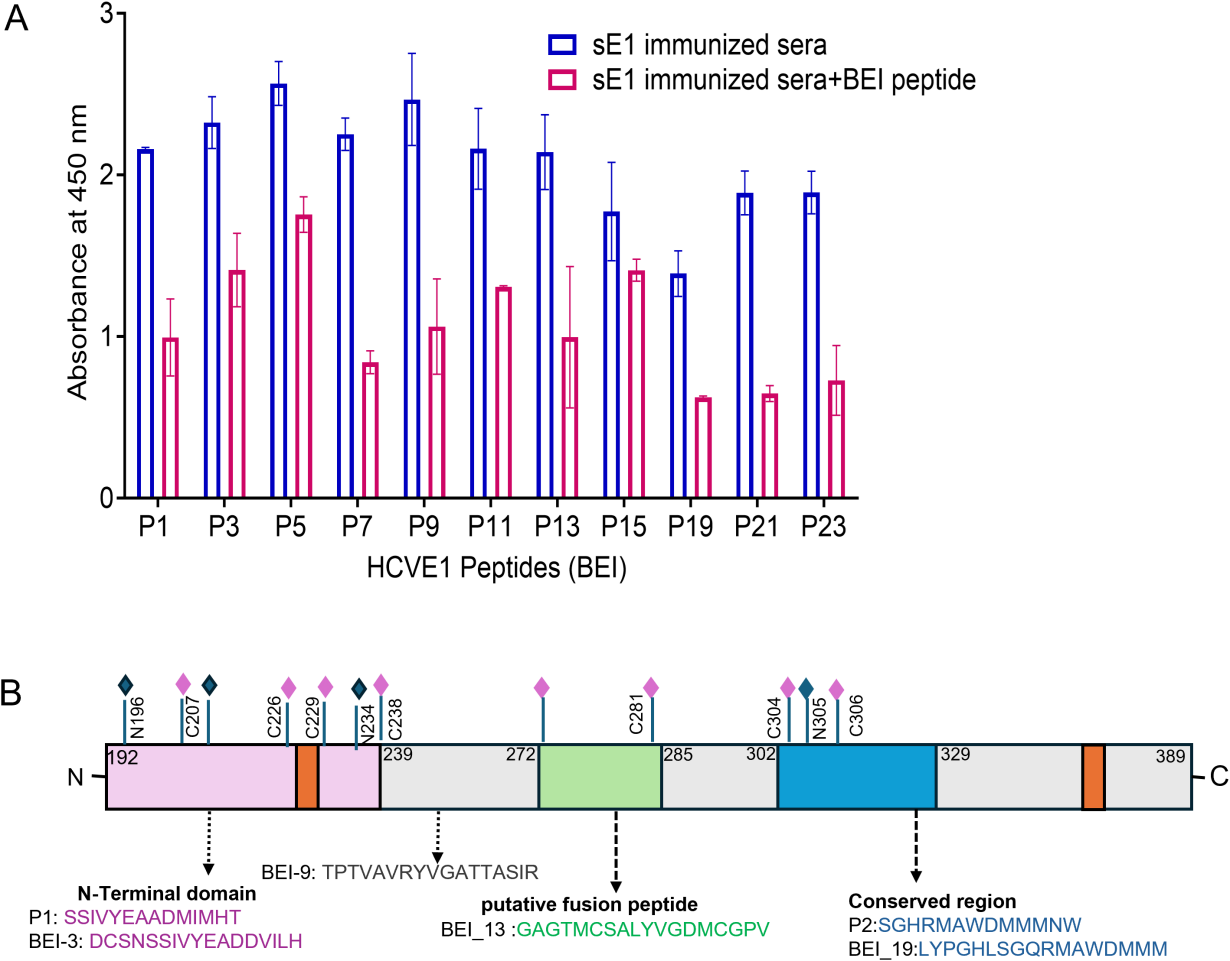
Linear peptide epitope recognition by sE1-immunized mouse sera and functional organization of the epitopes on E1. **(A)** Linear peptide epitope recognition by sE1-immunized mouse serum antibodies. Values are expressed as mean ± SD. **(B)** Schematic presentation of the HCV E1 protein showing functional domains and the positions of the selected peptides.

A different peptide, BEI peptide 13 (amino acid residues 269–282), corresponds to the putative fusion region and also showed binding specificity, while BEI peptide 19 (aa residues 314–324) corresponds closer to the C-terminal domain of E1. The identified two linear B-cell epitopes, P1 (aa residues 210–233) and P2 (aa residues 315–327), aligned with BEI peptides 3 and 19 of HCV genotype 3, respectively. Validating their potential relevance among the overlapping peptide clusters, strong binding was displayed for peptide 3, followed by peptide 19 (**Figure 1A**). We also performed ELISA inhibition using the pooled antiserum preincubated with the test peptide and subsequently added to the E1 antigen-coated ELISA plate. A significant reduction in the antibody binding signal of the antiserum was observed, indicating competition of the peptides (**Figure 1A**). The E1 (genotype 1a) mouse sera showed cross-reactivity even with the variable BEI sequences. For example, peptides 1/2/3 (N-terminal residues) and 7/8/9 (characteristics summarized above) contained two strong binding epitopes, although the peptides appeared to be closely located and contained variable amino acid residues. Based on the results, we grouped the peptides into four categories (**Table 1**): Group A, P1 and BEI peptide 3; Group B, BEI peptide 9; Group C, BEI peptide 13; and Group D, P2 and BEI peptide 19. Peptide-specific antibody recognition was further verified and schematically presented (**Figure 1B**). BEI peptide 5 was excluded from the group, as it exhibited good binding specificity, but lower inhibitory activity compared to the other peptides.

**Table 1:** List of peptides identified in initial screening with corresponding known characteristics.

Peptide	Sequence	Peptide position	Functions	Reference
Group A				
P1	SSIVYEAADMIMHT	210–223	Significantly inhibit the interaction of E1 with apolipoproteins, I212, and H222 cross-talk with E2	Mazumdar et al., (17); Wahid et al., (43); Haddad et al., (8)
BEI peptide 3	DCSNSSIVYEADDVILH	206–223		
Group B				
BEI peptide 9	TPTVAVRYVGATTASIR	244–260	R260 neutralization 1a, 1b	Colbert et al., (23)
Group C				
BEI peptide 13	GAGTMCSALYVGDMCGPV	269–282	272–285 is fusion peptide region	Tong et al., (46)
Group D				
P2	SGHRMAWDMMMNW	315–326	H316–CLDN1 binding, M323, W326–SR-B1 interplay	Moustafa et al., 2018, (42)
BEI peptide 19	LYPGHLSGQRMAWDMMM	314–324		

Examination of a panel of 38 HCV-infected human sera via ELISA displayed ~44% reactivity to P1 peptide and ~92% reactivity to P2 peptide (16). Rabbit antiserum generated to P1 (SLU22) or P2 (SLU23) was reactive in ELISA and neutralized E1/E2 HCV pseudotype generated from genotype 1a (clone H77). Mouse sera from immunized sE1-mRNA-LNP were also tested via competitive ELISA for reactivity with the peptides spanning the N-terminus of E1 (aa residues 192–352 ectodomain) from a heterologous HCV genotype 3a (BEI Resources). Other investigators identified potential B-cell epitopes encompassing amino acids 309–325 and 321–338. A relatively weak binding capacity was noted with genotype 3a peptide, and this may be due to amino acid mismatches with the immunizing genotype 1a sequence. BEI peptides 2 and 7 showed a reduction in competitive ELISA with mouse antiserum to purified E1 protein. BEI peptides 13, 19, 21, and 23 displayed a weak reduction in reactivity and may imply that E1 overall contains a narrow range of B-cell epitopes as compared to the E2 protein. Together, these observations indicated the presence of both conserved and cross-reactive linear B-cell epitopes within the E1 protein and may contribute to antibody-mediated protection for broader HCV genotypes.

### 3.2 *In silico* verification of E1-specific linear B-cell epitopes

We verified after preliminary screening whether the four groups of the identified peptides bind via ELISA to fulfil the criteria of a potential B-cell epitope. In our earlier study, we characterized the antigenic sites of HCV E1 glycoprotein by superimposing the predicted secondary structure with the parameters to identify the epitopes localized in hydrophilic, flexible, and β-turn regions with a high probability of surface exposure. We predicted the potential linear B-cell epitopes using a computational approach as P1, located in a variable non-glycosylated region, and P2, positioned within a relatively conserved hydrophilic domain (16). P1 represented relatively conserved amino acid sequences among the HCV genotypes, while P2 represented a highly conserved region. In the present study, we predicted antigenicity of the four BEI peptides (3, 9, 13, and 19) for predicting epitopes from the IEDB in the modern sense of deep learning or machine learning based on an algorithm using physicochemical properties. To predict linear B-cell epitopes, we used the BepiPred 2.0 tool available through the IEDB analysis resource (http://tools.iedb.org/bcell/). BepiPred 2.0 employs a random forest machine learning algorithm trained on experimentally validated antibody antigen protein structure to identify potential linear B-cell epitopes. We analyzed peptides from Groups A to D for potential epitopes (**Table 2**). Residues with a prediction score ≥ 0.5 were considered likely to constitute a linear B-cell epitope. Among the four groups, BEI peptide 3 (Group A) exhibited a high-scoring region with the motif IVYEADD, showing a score consistently above the threshold for potential B-cell epitope activity. Similarly, BEI peptide 9 (Group B) showed epitope potential with the VGATT motif. These predicted motifs represented a candidate region for antibody binding, may contribute to the observed immunogenicity, and will need further experimental validation. We used BepiPred-2.0 from DTU (Department of Health Technology) for further confirmation, and we predicted that the amino acid sequences of BEI peptide 3 (SSIVYEADDV), BEI peptide 9 (RYVGATT), and BEI peptide 13 (ALYVGD) represent potential B-cell epitopes. We hypothesize that, combining the results from our previous and current studies, Groups A to D possess potential B-cell epitope characteristics. Notably, the BEI peptide 19 B-cell epitope prediction score was below 0.5 by BepiPred 2.0.

**Table 2:** *In silico* prediction of linear B-cell epitope in identified peptides.

BEI_3 (DCSNSSIVYEADDVILH)				BEI_9 (TPTVAVRYVGATTASIR)			
Position	Residue	Score	Assignment	Position	Residue	Score	Assignment
0	D	0.226		0	T	0.19	
1	C	0.284		1	P	0.243	
2	S	0.344		2	T	0.305	
3	N	0.397		3	V	0.36	
4	S	0.454		4	A	0.409	
5	S	0.486		5	V	0.45	
6	I	0.504	E	6	R	0.473	
7	V	0.526	E	7	Y	0.492	
8	Y	0.533	E	8	V	0.503	E
9	E	0.531	E	9	G	0.514	E
10	A	0.531	E	10	A	0.523	E
11	D	0.528	E	11	T	0.516	E
12	D	0.52	E	12	T	0.516	E
13	V	0.463		13	A	0.467	
14	I	0.395		14	S	0.41	
15	L	0.342		15	I	0.358	
16	H	0.277		16	R	0.293	

### 3.3 Conservation of the identified B-cell epitopes and cross-genotype recognition

Conservancy of the identified peptides was examined using the IEDB epitope conservancy analysis tool across the seven HCV E1 isolates (**Table 3**). Among the four epitope groups (Groups A–D), the highest level of conservation was observed in P2 and peptide 19 (BEI Resources), showing 92% sequence identity across six isolates and 100% identity with isolate 1a H72. Notably, amino acid stretches 316 to 327 were found to be 100% conserved across all seven isolate variations. The P2 epitope region had previously been reported by us (16), and monoclonal antibodies IGH526 and IGH505 surrounding this region were developed (32). The second most conserved epitope was identified in Group A (P1 and BEI peptide 3), which showed greater than 82% conservation across HCV genotypes, except for isolate 4.2.2. The motif SSIVYA within this region was 100% conserved in all the isolates, except 1b58. The 100% conservancy is meant specifically for the epitope “SSIVYE”. Genotype 5 exhibited an overall conservancy of ~71% across the 14-amino acid long peptide (**Table 3**). Group C, peptides that consisted of BEI peptide 13, showed moderate conservation of 72% in isolate 1a and approximately 66% across most other isolates, with 4.2.2 showing the lowest conservation of 61%. This Group C region corresponds to a functionally important domain of E1, previously reported as essential for viral entry and morphogenesis (33). A conserved E1 hydrophobic sequence (aa residues 272–281, CSALYVGDLC) is proposed to be a putative fusion peptide (pFP). The key residues C272, G278, D279, L280, and C281 are highly conserved among HCV genotypes, indicating a potential role in viral function. The interplays between E1 and E2 may play a critical role in HCV fusion (34, 35). Interestingly, a reactive cluster of amino acid residues 269–277 of HCV (Hutchinson) was identified earlier from antibody response in patient serum late after infection (36). However, well-documented neutralizing antibodies have not been reported to target the E1 fusion peptide. This may be due to the inaccessibility of the region for existing as masked within the E1–E2 heterodimer on the mature virion. Finally, Group B (BEI peptide 9) demonstrated the least sequence conservation across the tested HCV isolates, with 100% identity in genotype 3a, 58% in isolate 4.2.2, and only 47% in isolates 1a, 1b, and 5.2.1. However, within this peptide, the amino acid motif TPT was 100% conserved across all isolates, suggesting that it may represent a minimal but conserved epitope of potential interest for further study.

**Table 3:** Conservancy analysis of identified HCV E1 linear epitopes.

HCV isolates	Group A: P1 (SSIVYEAADMIMHT) (210–223)	Percentage of conservancy	Group A: BEI_3 (DCSNSSIVYEADDVILH) (206–222)	Percentage of conservancy	Group B: BEI_9 (TPTVAVRYVGATTASI R) (243–259)	Percentage of conservancy	Group C: BEI_13 (GAGTMCSALYVGDMCGP V) (267–284)	Percentage of conservancy	Group D: P2 (SGHRMAWDMMMNW) (315–327)	Percentage of conservancy
1a H77	SSIVYEAADAILHT	85.71	DCPNSSIVYEAADAILH	82.35	TPTVATRDGKLPTTQL R	47.06	GSATLCSALYVGDLCGSV	72.2	TGHRMAWDMMMNW	92.31
1a H72	SSIVYEAADAILHT	85.71	DCPNSSIVYEAADAILH	82.35	TPTVATRDGKLPTTQL R	47.06	GSATLCSALYVGDLCGSV	72.2	SGHRMAWDMMMNW	100
1b 58	ASIVFEAADMIMHT	85.71	DCSNASIVFEAADMIMH	70.59	TPTLAARNASVPTTTIR	47.06	GAAAFCSAMYVGDLCGSV	66.7	TGHRMAWDMMMNW	92.31
1b 34	SSIVYEAADMIMHT	100	DCSNSSIVYEAADMIMH	82.34	TPTLAARNASVPTTTIR	47.06	GAAAFCSAMYVGDLCGSV	66.7	TGHRMAWDMMMNW	92.31
3.1.2	SSIVYEADDVILHT	78.57	DCPNSSIVYEADDVILH	94.1	TPTVAVRYVGATTASI R	100	GAATMCSALYVGDACGAV	66.7	TGHRMAWDMMMNW	92.31
4.2.2	SSIVYEADHHILHL	64.29	DCPNSSIVYEADHHILH	82.35	TPTVAAPYIGAPLESLR	58.82	GTATACSALYIGDLCGGLV	61.1	TGHRMAWDMMMNW	92.31
5.2.1	SSIVYEANDLILHA	71.43	DCPNSSIVYEANDLILH	82.35	TPTLSAPSLGAITAPLR	47.06	GGAALCSALYVGDACGAV	66.7	TGHRMAWDMMMNW	92.31

### 3.4 Difference in epitope-specific reactivity of sE1 immunized mouse sera

**Figure 2:**
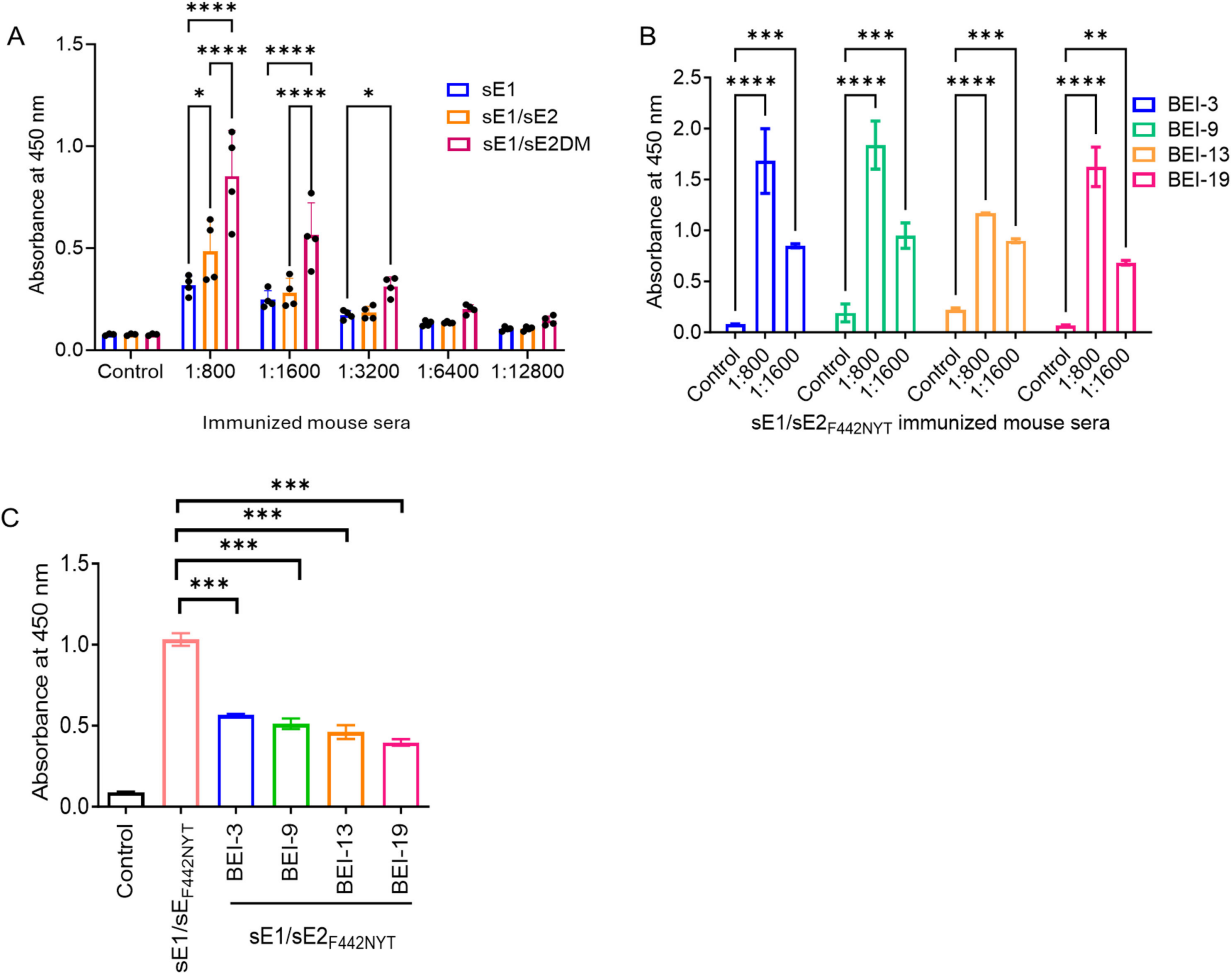
HCV E1-specific antibody response of immunized mouse antibodies. **(A)** ELISA of serially diluted pooled mouse sera following sE1, sE1/sE2, and sE1/sE2_F442NYT_ (double mutant or DM) immunization to purified E1-coated wells. **(B, C)** Linear peptide recognition by antibodies from sE1/sE2_F442NYT_ immunized mice. Results are shown as mean ± SD (n = 3). Statistical significance was determined by two-way ANOVA for panels A and B and one-way ANOVA for panel C and marked as *p < 0.05, **p < 0.005, ***p < 0.001, and ****p < 0.0001.

It was examined whether immune mouse sera raised against sE1, sE1/sE2, or sE1/sE2_F442NYT_ show a difference in antibody response to E1 epitope recognition. For this, ELISA was used to compare binding and to identify the immunodominant epitope targeted in a specific group of immunized mouse sera. E1 protein immobilized on a 96-well plate was incubated with a serial twofold dilution (1:800–1:12,800) of pooled immune mouse sera (**Figure 2A**). Interestingly, a difference in reactivity was noted from the binding intensity of sera in the following order: sE1/sE2_F442NYT_ > E1/sE2 or sE1.

We examined for preferential binding of sE1/sE2_F442NYT_ immunized sera to the peptides and observed a relative binding order of BEI peptide 9 > 3 or 19 > 13 (**Figure 2B**). The results suggested that BEI peptides 9, 3, and 19 encompass a stronger immunodominant region, whereas BEI peptide 13 is relatively less immunogenic. BEI peptides 3 and 19 correspond to the P1 and P2 epitope regions, respectively. In addition, there may be conformation epitope-dependent differences for E2 binding of the antibodies. Competitive ELISA using four selected BEI peptides (3, 9, 13, and 19) was used to investigate the specificity of antibody response (**Figure 2C**). Incubation of sE1/sE2_F442NYT_ immunized serum with the peptides before addition to the E1-coated plate significantly reduced binding. Together, the results support the role of the B-cell epitopes as key immunogenic determinants on the HCV E1 protein.

### 3.5 HCV pseudoparticle neutralization by rabbit antiserum to P1 or P2 peptide

**Figure 3:**
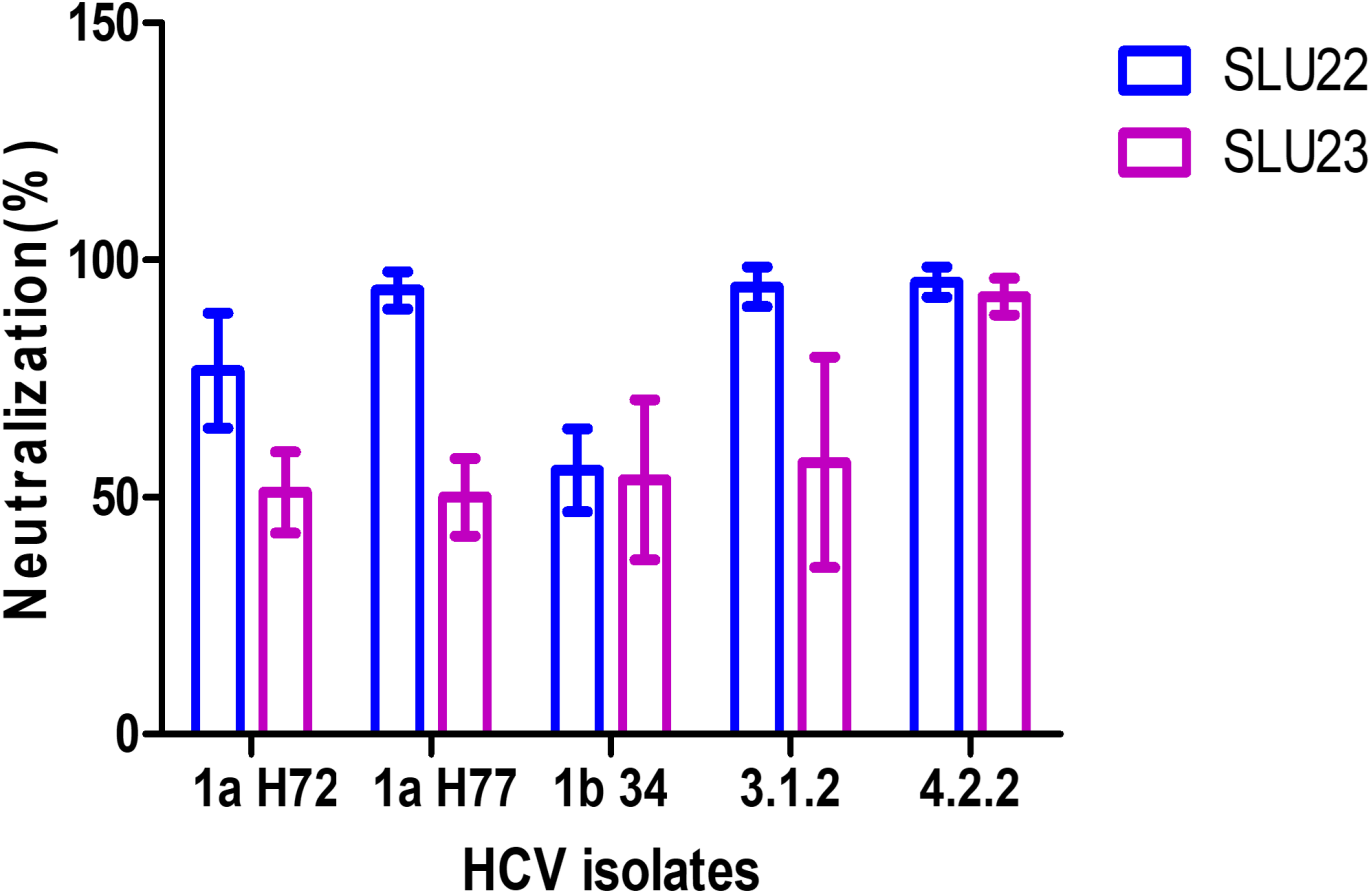
Neutralizing antibody response induced by peptide-immunized rabbit antibodies. Comparative neutralization of HCV pseudoparticles (HCVpp) from different isolates by SLU22 and SLU23 is shown. Values are expressed as mean ± SD (n = 3).

The peptides were identified as Group A (P1 or BEI peptide 3) and Group D (P2 or BEI peptide 19), containing the B-cell epitopes, based on the above results and from our previous studies. Whether the antibodies can functionally prevent HCV entry was determined, a key measure for protective immunity and vaccine efficacy. HCVpp neutralization assay was performed using lentivirus-derived pseudoparticles expressing envelope glycoprotein from five different HCV isolates (1aH77, 1aH72, 1b34, 4.2.2, and 3.1.2). A serial dilution of SLU22 or SLU23 antiserum was incubated with the respective HCVpp (10^3^ or 10^4^ RLU), and the neutralizing effect was assessed by comparing the infection level in the presence or absence of immune serum and untreated controls (**Figure 3**). SLU22 demonstrated broad and potent neutralization activity, achieving >80% inhibition of infectivity against HCV isolates 1aH72, 1aH77, 3.1.2, and 4.2.2. In contrast, SLU23 showed 55% neutralizing activity of pseudotypes generated from HCV isolates 1aH72, 1aH77, and 3.1.2, while >85% neutralization was observed with 4.2.2. The other immunogenic regions of E1 spanning B-cell epitopes may elicit non-neutralizing antibodies irrespective of the known roles in HCV biology or host immune function (37).

### 3.6 The role of E1 in attachment and binding for virus entry

**Figure 4:**
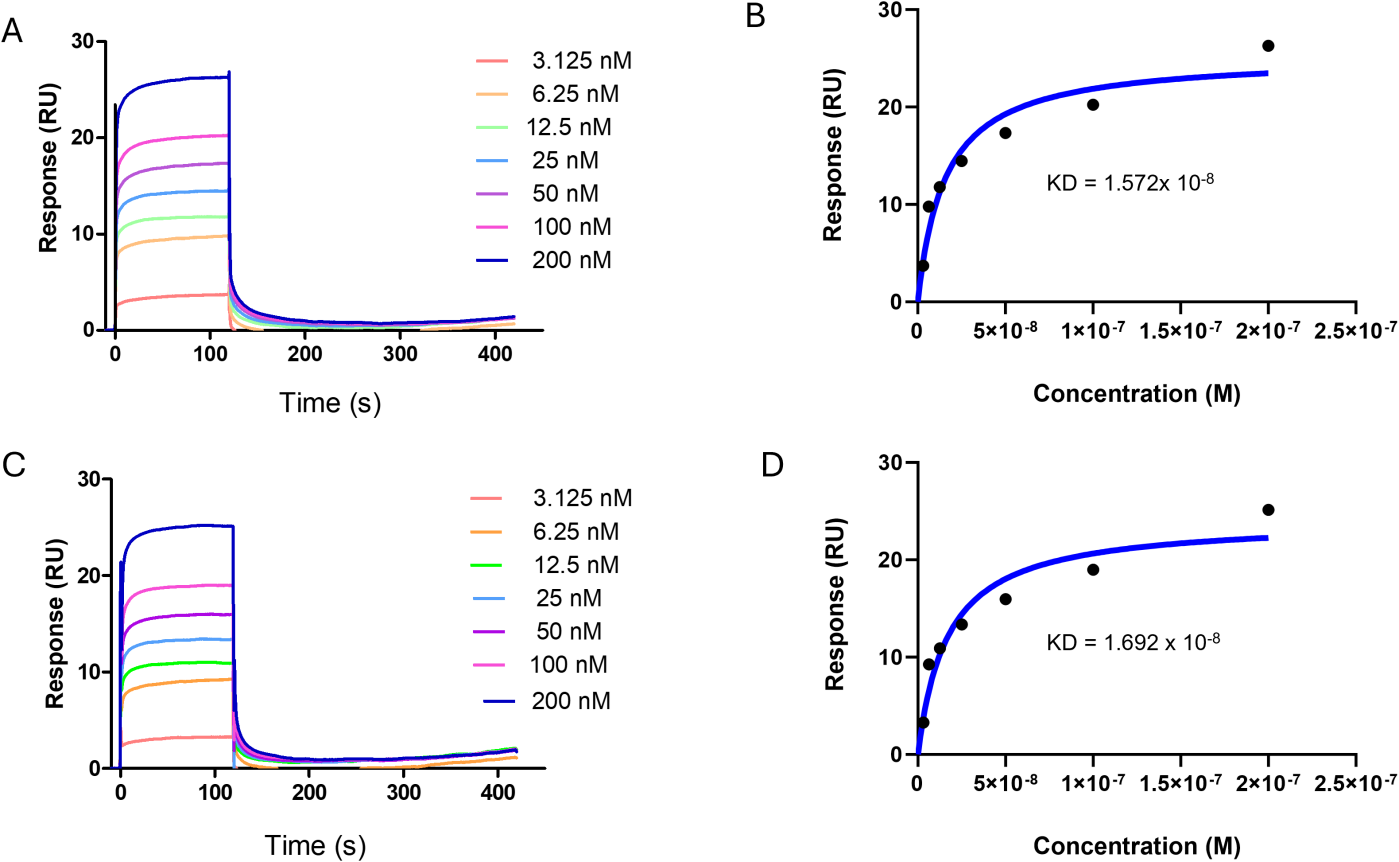
Binding kinetics of E1E2 to peptide-immunized rabbit serum antibodies. **(A, C)** Surface plasmon resonance (SPR) was used to evaluate the interaction of E1E2 with the Fab surfaces of SLU22 and SLU23 immobilized on a CMD200M SPR sensor chip using a Biacore S200. Representative fitted SPR sensorgrams are showing the association and dissociation phases (response vs. time) for SLU22 and SLU23, respectively. **(B, D)** Equilibrium binding analysis displaying response curves as a function of increasing analyte concentration with constant immobilized Fabs of SLU22 and SLU23. The equilibrium dissociation constants (KD) were determined as ~1.57 × 10^−8^ M for SLU22 and ~1.69 × 10^−8^ M for SLU23.

The binding kinetics of HCV1a H77 enveloped protein E1E2 with the Fab fragments of SLU22 and SLU23 were investigated using SPR spectroscopy. The interaction shows very rapid antigen binding, occurring within 1 s, followed by dissociation over 115 s. From the equilibrium analysis (concentration Vs response), the equilibrium dissociation constant (KD) was estimated to be 1.57 × 10^−8^ M for SLU22 and 1.69 × 10^−8^ M for SLU23 (**Figure 4**). In contrast, kinetics analysis (time Vs response) provided KD values of 2.15 and 1.73 nM. The kinetic parameters’ association rate constants (*ka*) were estimated as 1.34 × 10^2^ M^−1^ s^−1^ for SLU22 and 1.82 × 10^2^ M^−1^ s^−1^ for SLU23, and the dissociation rate constants (*k off*) were 9.60 × 10^6^ s^−1^ and 6.93 × 10^7^ s^−1^, respectively. Together, the findings indicated that both SLU22 and SLU23 bind HCV glycoprotein with high affinity.

**Figure 5:**
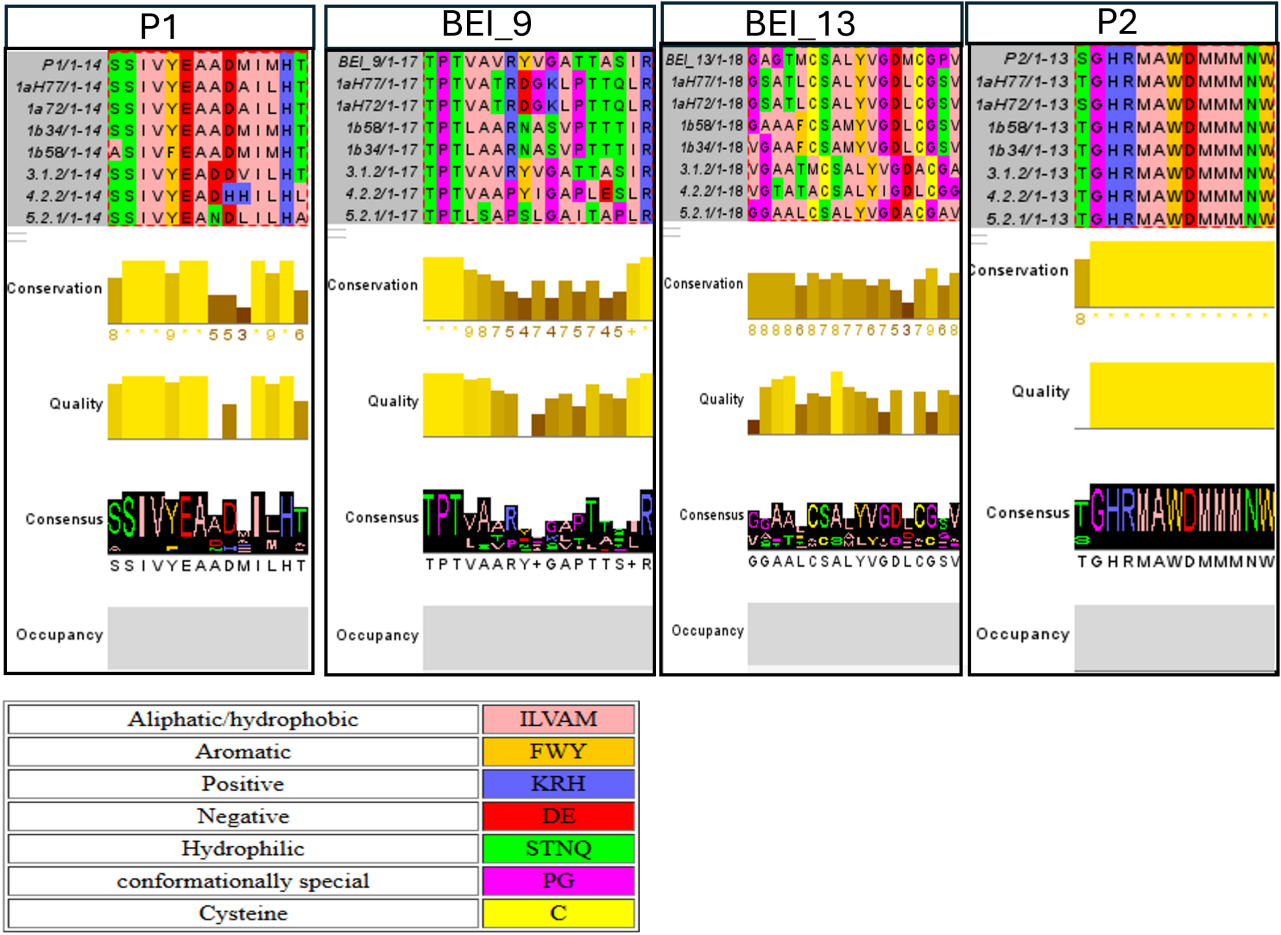
Comparative sequence alignment of the identified peptide across HCV genotypes. Multiple sequence alignment was performed in Jalview. Zappo coloring highlights amino acid chemical characteristics (hydrophobic, aromatic, positive, negative, hydrophilic, conformationally special, and cysteine residues). The consensus sequence and conservation plot are shown below the alignment. The light-yellow bars represent higher sequence conservation across genotypes, while the darker bars indicate variability.

Furthermore, we examined the comparison of the biophysical characteristics of the aligned sequences, such as amino acid conservation, and visualization of physicochemical properties using the Jalview software. The analyses helped to highlight the regions where sequences share similar biochemical traits, not just identical residues. Thus, we generated a Jalview multiple sequence alignment with Zappo coloring and conservation/consensus plot (**Figure 5**). The P2 peptide has the most conserved region, GHRMAWDMMMNW, among the other sequences. The P1 and BEI peptide 3 plot showed that SSIVYEA is well conserved across the genotypes; BEI peptide 13 contains YVGD, while BEI peptide 9 represents the conserved TPT region. Comparison of physicochemical characteristics revealed the presence of amino acid features consistent with previously described B-cell epitopes. Amino acid frequency analysis indicated that some of the residues are more frequent than others, such as serine (8.3%), alanine (7.75%), leucine (7.87%), and glycine (7.45%). In contrast, cysteine (1.56%), tryptophan (2.24%), and methionine (2.37%) are less frequent. However, when comparing SWISS-PROT data, tryptophan, proline, and histidine are clearly overrepresented in B-cell epitopes (38). Other studies have shown that tyrosine and tryptophan are significantly overrepresented in epitopes, while valine is significantly underrepresented (39, 40). The B-cell epitopes typically contain a hydrophobic core flanked by charged amino acids based on spatial amino acid composition. Several of these previously described B-cell epitope features are consistent with our identified peptides. The P1 peptide contains serine, alanine, histidine, tyrosine, and leucine and frequently expresses B-cell epitope residues. The peptide also includes a hydrophobic region flanked by charged and hydrophilic amino acids. Similarly, P2 has a hydrophobic region flanked by hydrophilic or charged residues and includes glycine, alanine, histidine, and tryptophan. BEI peptide 9 contains conformationally special amino acid proline and matches the spatial amino acid composition of the hydrophobic core with flanking hydrophilic regions. BEI peptide 13 contains serine, tyrosine, alanine, and leucine, which are frequently found in B-cell epitopes.

### 3.7 Inhibition of E1/E2 interaction with Huh7.5 cells by P1- or P2-specific antiserum

**Figure 6:**
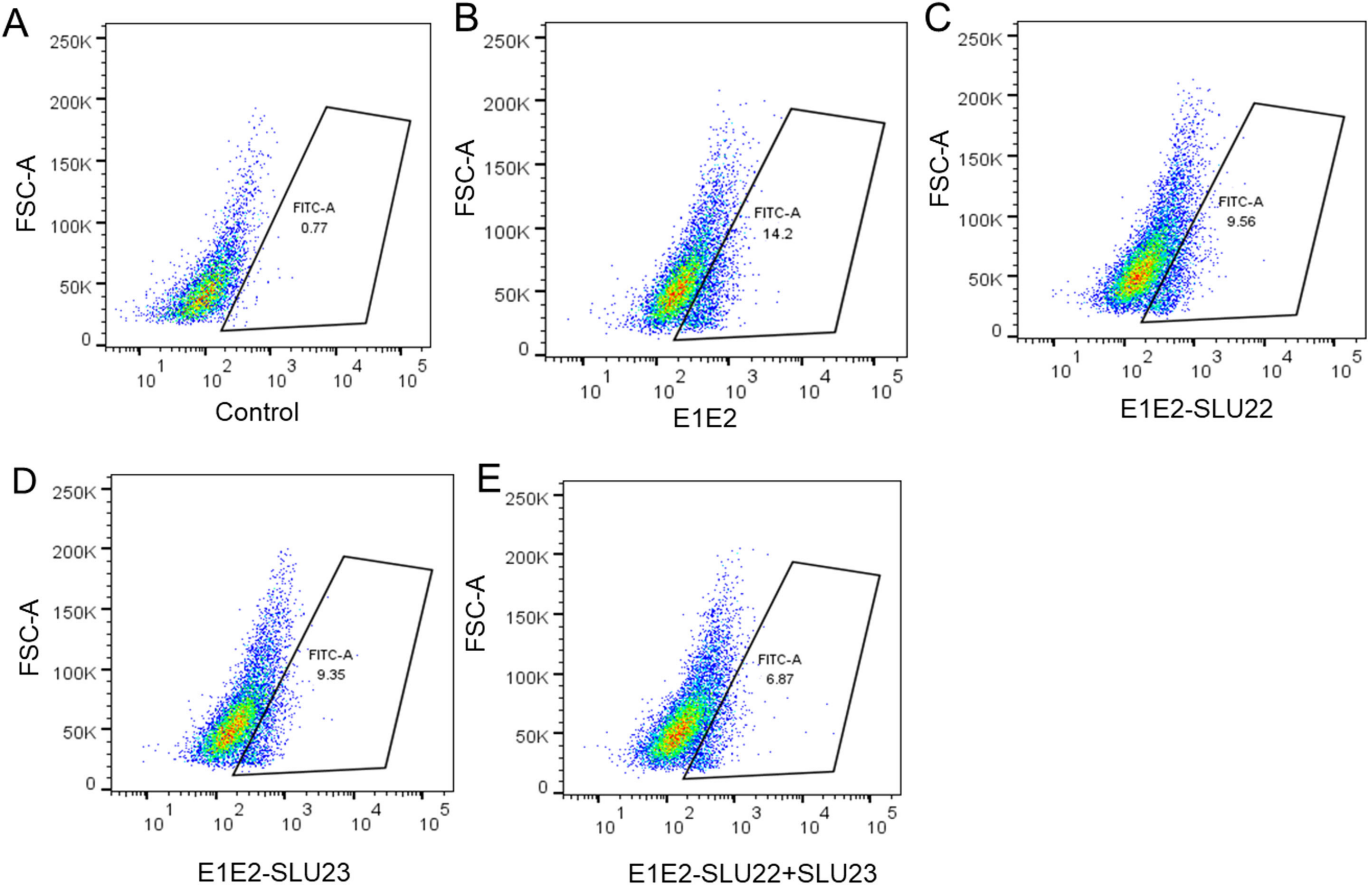
Binding of peptide-immunized rabbit serum to purified E1/E2 and its inhibitory effect on receptor interaction. HCV E1/E2 was preincubated with rabbit serum raised against peptide P1 (SLU22) or P2 (SLU23) and in combination. The receptor interaction on Huh 7.5 cells by the antigen–antibody complex was analyzed via flow cytometry. **(A)** Untreated control. **(B)** E1 and E2. **(C)** E1, E2, and SLU22. **(D)** E1, E2, and SLU23. **(E)** E1, E2, SLU22, and SLU23.

The E2 envelope protein of HCV contains several key receptor-binding domains, while E1 perhaps plays a supportive role in modulating E2 host cell surface receptor interactions and in facilitating membrane fusion as a critical step for virus entry (41). Loss of the key E2 domain disrupts the receptor binding and membrane association. However, E1 is required for proper folding of the E1/E2 complex and receptor engagement for efficient infection to begin the virus life cycle. The broad or cross-genotype HCVpp neutralization observed with antiserum to P1 or P2 peptide prompted us to study how antibodies to these regions influence the E1/E2 association in binding to cell surface receptors (CD81 and SR-B1). For this, the binding of E1/E2 to Huh 7.5 cell surface was investigated in the presence or absence of SLU22 and SLU23 and analyzed via flow cytometry using FITC-conjugated E2-specific AP33 monoclonal antibody. Purified E1/E2 proteins were first incubated with the antibody SLU22 or SLU23 at a 1:10 dilution for 2 h at 37°C, followed by the addition to Huh7.5 cells. Flow cytometry results revealed that E1/E2 binding to Huh7.5 cells was 14.2%, which reduced to 9.5% in the presence of SLU22 and 9.35% in the presence of SLU23 (**Figure 6**). The binding was further decreased to 6.87% when both antisera were present. These results indicated that P1 and/or P2 immunized rabbit antiserum significantly inhibited the E1/E2 binding to Huh7.5 cell surface receptors, highlighting their potential role in neutralizing virus entry.

**Figure 7:**
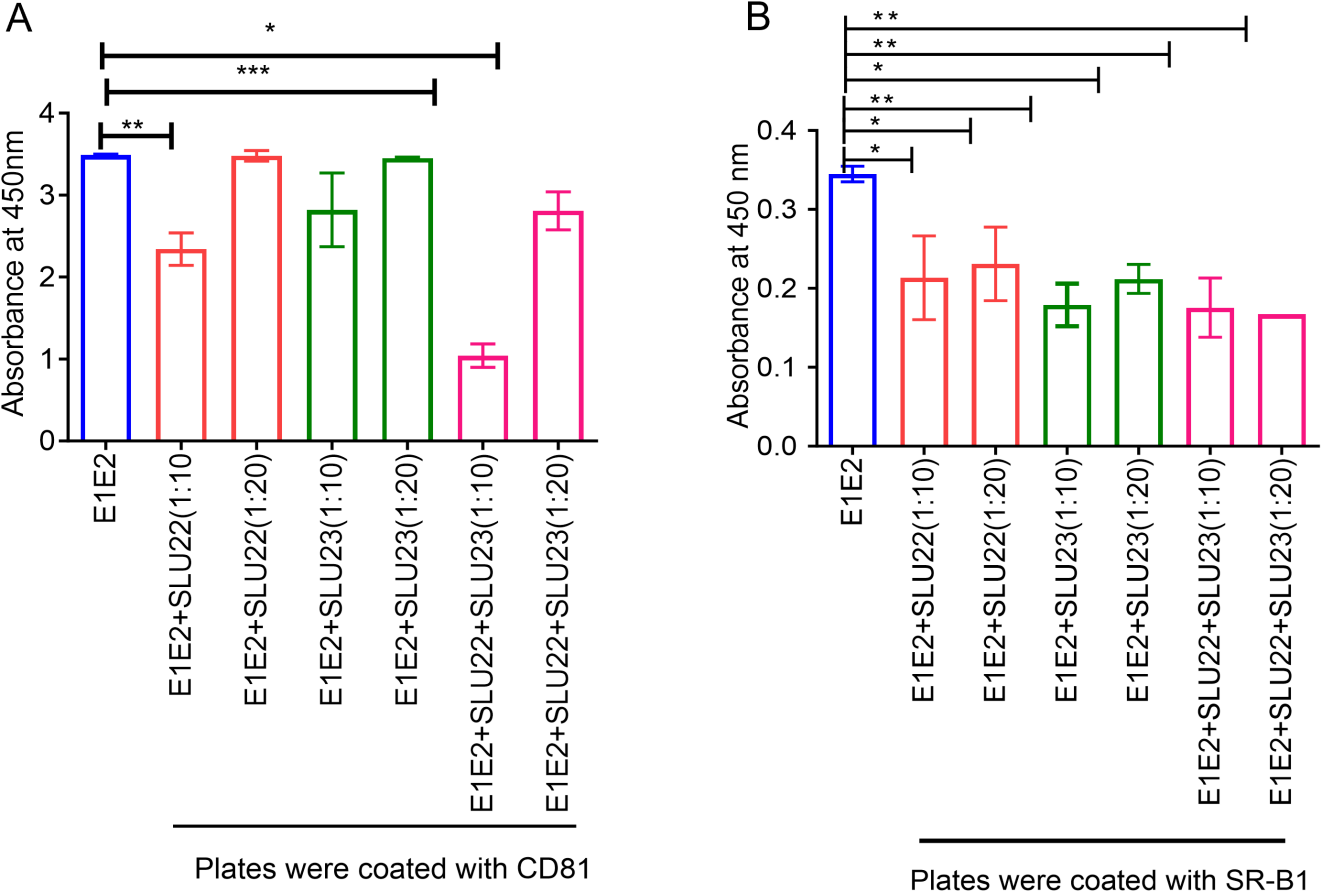
Binding of peptide-immunized rabbit sera to the purified E1/E2 and inhibition of CD81 and SR-B1 interaction. **(A)** Inhibition of E1/E2 binding to CD81 by rabbit antisera raised against peptide P1 (SLU22), peptide P2 (SLU23), or their combination. **(B)** Inhibition of E1/E2 binding to SR-B1 by rabbit antibodies. Preincubation of E1/E2 with antiserum was performed before evaluation on CD81- or SR-B1-coated plate. The results are presented as the mean ± SD (n = 3). Statistical significance was determined by two- or one-way ANOVA and marked as *p < 0.05, **p < 0.005, and ***p < 0.001.

We subsequently focused on the effect of P1- and/or P2-specific antibodies on HCV envelope glycoprotein(s) for CD81, followed by SR-B1. Monoclonal antibody AP33 recognizes a conserved linear epitope on the E2 (amino acid residues 412–423, QLINTNGSWHIN). The CD81 receptor binds to E2 at the large extracellular loop, with its binding region mapped to E2 aa residues 412–446. The critical determinant for CD81 binding is W420, which plays a pivotal role in stabilizing the interaction between E2 and the large extracellular loop of CD81, and is also essential for AP33 binding. The CD81 binding study (**Figure 7A**) showed that SLU22 antiserum had a modest inhibitory effect on E1/E2 binding as compared to the SLU23 antiserum. The combination of SLU22 and SLU23 also showed a modest synergistic inhibition of E1/E2 binding to CD81 and may be due to the impact on the conformational region. The SR-B1 binding assay showed that rabbit antiserum to P2 exhibited a strong inhibitory effect on SR-B1 as compared to P1 antiserum (**Figure 7B**). A combination of the antisera also demonstrated an inhibitory effect, although a synergistic or additive effect was not observed for the inhibition of SR-B1 binding. HCV E1 and E2 interplay is important in the HCV life cycle. In a mature virus, E1 may associate with E2 for a functional conformation for interaction with cellular receptors (42). Mutation of each of the eight conserved cysteines of E1, as well as some residues in the N-terminal part of E1 (JFH1 I212, T213, H222, and W239), may affect the interaction of E2 with CD81 as the primary HCV receptor (43, 44). Similarly, several mutations in the residues of the E1 α2 region may affect the dependence of HCV on SR-B1 (43).

## 4 Discussion

HCV E1 envelope glycoprotein induces linear B cell-specific antibodies and T cell-associated antiviral responses (5, 16, 45). Molecular and structural analyses of E1 and E2 have advanced our understanding of the functional regions of HCV envelope glycoproteins. The studies have linked the importance of the conserved E1 amino acid residues with the HCV life cycle (42). Our current study provided novel insights into the immunogenic landscape of HCV E1 glycoprotein. In our previous work, we demonstrated that immunization with the sE1-mRNA-LNP candidate vaccine elicited a higher neutralization antibody titer compared to sE2 or sE1/sE2. Immunization with sE1 also induced T cell-associated cytokine response and protection against HCV surrogate challenge infection in a mouse model. Our major observations from the present study include the following. i) Identification of the four linear B-cell epitopes located on E1. Two of the epitopes are relatively or highly conserved (P1 and P2, respectively), generating a strong binding antibody response in immunized rabbits. The P1 is localized in the α1 helix region, closer to the N-terminal ectodomain of the envelope E1/E2 major CD81 receptor-binding area. However, P2 is localized in residues reported to interplay with the tight junction protein CLDN1 and SR-B1. ii) Rabbit antibodies to the P1 and P2 epitopes showed cross-reactivity and broad neutralization activity to four HCV genotypes, underscoring them as potential pan-genotypic vaccine components. iii) Preincubation of HCV E1/E2 with rabbit anti-peptide sera individually and in combination exhibited Huh 7.5 cell surface receptor recognition and suggested potential for the induction of conformational changes of the E1/E2 complex, thereby inhibiting the ability to engage with host cell surface receptors. iv) The results revealed that preincubation of E1/E2 with SLU22 reduced CD81 binding, while a combination of SLU22 and SLU23 exerted a synergistic inhibitory effect on binding. In contrast, SLU23 alone specifically inhibited E1/E2 interaction with SR-B1. Thus, E1 linear epitope-specific rabbit antisera display inhibitory CD81 or SR-B1 binding function of the E2 chaperone protein in association as E1/E2 complex. v) Mice immunized with sE1, sE1/E2, or sE1/E2_F442NYT_ contributed to antibody generation recognizing E1 epitopes when presented alone. In contrast, P1 epitope recognition was weak when mice were immunized together with unmodified E2 and may reflect a negative immune regulatory role. Sera from sE1/E2_F442NYT_ immunized mice exhibited a stronger binding as compared to the other selected antigens.

A critical cross-talk between E1 and E2 is proposed to modulate E1/E2 binding to HCV entry receptors CD81 and SR-B1 (34), where E1 is likely assisting E2 by maintaining a functional conformation (46). Specific amino acid sequences located on the P1 region of E1 (residues I212 and H222) are reported to cross-talk with E2 (42). We showed preferential association of E1 N-terminal ectodomain with ApoE and ApoB. HCV E1 is also shown to directly bind CD36 to facilitate virus attachment (47) and may involve LDL-R for virus entry. We previously reported a significant reduction in E1-G pseudotype plaque numbers (~70%) by inhibiting LDL-R ligand binding activity using human proprotein convertase subtilisin/kexin type 9 and platelet factor-4, while they had minimal effect on E2-G pseudotype (17). Our results suggested that antibody targeting the P1 epitope may effectively block or induce conformational change of the E1/E2 complex, diminishing the ability to bind to CD81.

Information in the literature suggests that only a limited function of E2 may depend on the association of E1, like interaction with CLDN, SR-B1, CD81, or putative fusion peptide activity (46), although the other predictive functions would require experimental support. A mutation D263A in E1 abolishes viral infectivity and leads to the secretion of viral particles devoid of genomic RNA (8). The deletion of the putative fusion peptide (pFP) has no effect on the E1E2 heterodimerization but completely abolishes the production and release of infectious virions (48). HCV chimeric E1/E2 heterodimer was generated to identify residues involved in cross-talk between the two envelope proteins for a critical role in cell entry (34). Several amino acids on E1 were reported for cross-talk with E2. The functional characterization of the interaction indicated that a specific E1/E2 association may be involved in binding to the primary receptors and in membrane fusion, highlighting its bystander multifunctional role in HCV cell entry. However, computational prediction and experimental validation identified immunogenic T-cell epitopes from HCV E1 glycoprotein (45). Immunogenic potential of two specific peptides from the E1 glycoprotein (QVRNSSGLY and QLFTFSPRH) suggested involvement in multiple cytokine induction associated with both innate and adaptive immune functions from lymphocytes of patients after recovery from HCV infection. Our recent studies demonstrated that sE1 alone when used as a candidate vaccine from a mRNA-LNP platform exhibited distinct protective antigenic properties, inducing higher neutralization titers compared to similar sE2 or sE1/sE2 immunization. Additionally, sE1 promoted T cell-associated cytokine response (5).

Our experiments utilized HCV envelope glycoproteins from three different genotypes. Immunized mouse sera were directed to HCV genotype 1a (H77), purified virus proteins tested were procured from HCV genotype 1b *HC-J4* (Sino Biological), and most of the E1 peptides used were chosen from genotype 3a K3a/650 sequence (BEI). Differences in solubility of these peptides in the assay may make quantitative variations, although the qualitative nature of reactivity will likely remain similar. Our results indicated that the reactive E1 epitopes are present in the majority of these different HCV genotypes examined. The P1 region is relatively conserved, and rabbit antiserum (SLU22) can inhibit HCV E1 association with apolipoproteins for pseudotype virus entry inhibition, likely via LDL-R. The resulting inhibitory titer of SLU22 was modest (approximately 1/10) for pseudotype virus neutralization. Our previous results suggested that HCV anti-sE1 sera can induce a strong pseudotype virus neutralization titer (5, 17). Structural biology-related studies have suggested that the HCV E1/E2 protein complex is remarkably fragile and does not easily maintain function after single amino acid changes from a comprehensive functional screening (9). This interpretation lacks an answer for further understanding the biology of HCV persistence as quasispecies or in the presence of specific neutralizing and non-neutralizing antibodies in chronically infected humans.

Both the transmembrane and ectodomains of E2 are involved in the cross-talk with E1 for conformational changes and entry. E1 and E2 are suggested to possess domains responsible for fusion (49–51). Several antibodies directed against E1 were shown to neutralize cell entry, presumably at a stage distinct from receptor binding (7, 52, 53). E1 may indirectly play a role in HCV infection and may remain for further elucidation. We have shown HCV E1 protein undergoes fusion at low endosomal pH (54). VSV/HCV pseudotype virus was generated from the E1 or E2 ectodomain fused to the VSV G transmembrane and cytoplasmic tail (important for fusion). Treatment of HCV E1- or E2-derived VSV pseudotypes in media between pH 5 and 8 before adsorption on cells did not significantly reduce plaque numbers. However, treatment of cells with lysosomotropic agents or inhibitors of vacuolar H(+) ATPases had an inhibitory role in virus entry. Thus, the putative fusion peptide (FP) of E1 may be contributing to HCV biology (55).

## 5 Conclusion

Overall, these findings enhance a better understanding of the structural and functional attributes of E1 epitopes and support E1 cross-reactivity, broad neutralization, and its supportive role in HCV entry. These findings will be critical for guiding a rational design of HCV vaccines and antibody-based therapeutics.

## Data Availability

The original contributions presented in the study are included in the article/supplementary material. Further inquiries can be directed to the corresponding author.
